# Triple-Knockout, Synuclein-Free Mice Display Compromised Lipid Pattern

**DOI:** 10.3390/molecules26113078

**Published:** 2021-05-21

**Authors:** Irina A. Guschina, Natalia Ninkina, Andrei Roman, Mikhail V. Pokrovskiy, Vladimir L. Buchman

**Affiliations:** 1 School of Biosciences, Cardiff University, Cardiff CF10 3AX, UK; ninkinan@cardiff.ac.uk (N.N.); andrei.yu.roman@gmail.com (A.R.); buchmanvl@cardiff.ac.uk (V.L.B.); 2 Institute of Physiologically Active Compounds Russian Academy of Sciences (IPAC RAS), 1 Severniy Proezd, Chernogolovka 142432, Moscow Region, Russia; 3 Research Institute of Living Systems Pharmacology, Belgorod State National Research University, 85 Pobedy Street, Belgorod 308015, Belgorod Oblast, Russia; pokrovskii@bsu.edu.ru

**Keywords:** synucleins, triple-knockout mouse model, phospholipids, non-polar lipids, fatty acids

## Abstract

Recent studies have implicated synucleins in several reactions during the biosynthesis of lipids and fatty acids in addition to their recognised role in membrane lipid binding and synaptic functions. These are among aspects of decreased synuclein functions that are still poorly acknowledged especially in regard to pathogenesis in Parkinson’s disease. Here, we aimed to add to existing knowledge of synuclein deficiency (i.e., the lack of all three family members), with respect to changes in fatty acids and lipids in plasma, liver, and two brain regions in triple synuclein-knockout (TKO) mice. We describe changes of long-chain polyunsaturated fatty acids (LCPUFA) and palmitic acid in liver and plasma, reduced triacylglycerol (TAG) accumulation in liver and non-esterified fatty acids in plasma of synuclein free mice. In midbrain, we observed counterbalanced changes in the relative concentrations of phosphatidylcholine (PC) and cerebrosides (CER). We also recorded a notable reduction in ethanolamine plasmalogens in the midbrain of synuclein free mice, which is an important finding since the abnormal ether lipid metabolism usually associated with neurological disorders. In summary, our data demonstrates that synuclein deficiency results in alterations of the PUFA synthesis, storage lipid accumulation in the liver, and the reduction of plasmalogens and CER, those polar lipids which are principal compounds of lipid rafts in many tissues. An ablation of all three synuclein family members causes more profound changes in lipid metabolism than changes previously shown to be associated with γ-synuclein deficiency alone. Possible mechanisms by which synuclein deficiency may govern the reported modifications of lipid metabolism in TKO mice are proposed and discussed.

## 1. Introduction

The synucleins are small, highly conserved proteins, expressed primarily in the nervous system of vertebrates. The synuclein family comprises three closely related proteins, α-, β-, and γ-synucleins with distinctive functions that share sequence homology with other proteins, such as the class A2 lipid-binding domains of the apolipoproteins, 14-3-3 chaperones, and several small heat-shock-proteins [1,2,3,4,5].

Interest in this protein family is first and foremost associated with their special role in human neurodegenerative diseases, collectively termed “synucleinopathies”, and largely with a potential involvement of α-synuclein aggregates in Parkinson’s disease (PD). 

α-Synuclein was originally identified as a protein associated with synaptic vesicles (SV) [6]. It is widely expressed in various brain regions but localises specifically to the nerve terminal where it modulates synaptic functions. Several non-neural tissues, e.g., red blood cells also express this protein. Like α-synuclein, β-synuclein also localises at presynaptic terminals. β-Synuclein has been shown to inhibit the aggregation of α-synuclein both in vitro and in vivo [7,8]. Mutations in β-synuclein have been linked to a case of dementia with Lewy bodies [9,10]. Recently, there have been suggestions that β-synuclein can play a significant, pathophysiological role in etiology of PD through its interaction with dopamine metabolites [11]. In contrast to α- and β-synucleins, γ-synuclein is predominantly expressed in sensory neurons and motoneurons of the peripheral nervous system. It has not been associated with the onset or suppression of PD. This family member is also expressed in retina and in a variety of cancer cells [1,2]. 

α-Synuclein is a 140-amino-acid protein, and its sequence comprises three overlapping regions including the N-terminal region, the NAC (non-Aβ component) region and C-terminal region. The positively charged N-terminal region contains seven 11-residue repeats with the structural features similar to those of apolipoprotein-like class A2 amphipathic α-helix. 

This terminal also retains one of two conserved fatty acid binding protein (FABS) motifs. N-regions of β- and γ-synucleins show a high structural homology to that of α-synuclein [2]. β- and γ-Synucleins are distinct form α-synuclein by the lack of most NAC sequence [12,13,14]. These differences have been linked to the various propensities of synucleins to aggregation with α-synuclein having the highest proneness to form aggregates, while β-synuclein is the least prone [15]. The negatively charged C-terminal region is highly unstructured and has been found to remain unstructured in the amyloid fibril. Post-translation phosphorylation and nitration of some sites of this region change the net charge of the C-terminal region and affect the α-synuclein aggregation and membrane binding. Moreover, the C-truncated form of α-synuclein exacerbates an aggregation [1,2,16]. From these findings, it has been suggested that this region may play a role in regulation of α-synuclein function and conformation. Moreover, this tail mediates interactions between α-synuclein and soluble *N*-ethylmaleimide-sensitive factor attachment protein receptors (SNARE) complex proteins [17]. This domain is less conserved among α, β-, and γ-isoforms.

All members of the synuclein family interact robustly with lipid membranes and appear to be important for the physiological functions of proteins while influencing the pathological aggregation of α-synuclein. Many in vitro experiments have demonstrated the affinity of synucleins towards membranes containing negatively charged lipids, esterified with oleic, palmitoleic and polyunsaturated fatty acids, suggesting that it may specifically recognise the membrane microdomains that differs in fluidity and charging [8,18,19,20,21]. α-Synuclein also associates with the membranes composed of zwitterionic phospholipids with varied reports of strong or weak binding [22,23,24]. Upon membrane binding, synucleins can adopt a range of structural architectures** [19,25]. The α-synuclein helix has been shown to extend parallel to the curved membrane; its conserved N-terminal residues are attached to the twitterionic headgroups of the outer membrane leaflet, while uncharged residues penetrate the acyl chain region, ensuring a strong interaction of synuclein monomers and membrane bilayer [25,26,27,28]. Two other synucleins are also able to interact with lipid bilayers and generate membrane curvature [10,29]. This interaction induces a large scale of membrane remodeling, such as tubulation and vesiculation; it also may reorganise membrane bilayer through lifting and lateral expansion of lipids, leading to the membrane warping and structural destabilisation [27,30,31,32,33,34]. Moreover, oligomeric species formed during self-assembly of α-synuclein enhanced membrane permeability and induced substantial increases in the intracellular reactive oxygen species production and, thus, neuronal toxicity [15,35]. In contrast, aggregation of α-synuclein may be neuroprotective by sequestering its toxic oligomer. In spite of some controversy, the majority of findings support the connection between oligomeric α-synuclein and PUFA oxidation in dopaminergic toxicity which is increasing, when α-synuclein interacts with PUFA and their oxidation products. Insofar, many studies revealed the molecular basis of interactions between α-synuclein and PUFA and their importance in both, neuroprotection and neurodegeneration and in vivo, including many PD cell and animal models [19,36,37,38,39,40]. Lipids influence the structure and, consequently, the functions of synucleins (and vice versa, of note!) through complex interplaying and overlapping mechanisms. As an interplay, numerous recorded changes in lipids (and lipid metabolism in cells and various synuclein mouse models) were associated to either the synuclein loss-of-function or a toxic gain-of-function hypothesis [40,41,42,43,44]. In summary, they include: uptake of fatty acids into cells; changes in brain cardiolipin and its fatty acid acyl chains [2]; an increased level of brain triacylglycerol (TAG) content in α-synuclein mouse mutant model associated with increased fatty acid synthase expression and acyl-CoA synthase activity, with no variations in TAG lipase activity or in fatty acid β-oxidation [45]. α-Synuclein-null animals showed decreased palmitate uptake and the altered metabolism of this FA in brain, and turnover of arachidonate and docosahexanoate in brain phospholipids [46,47]. Our previous work on γ-synuclein null mice showed significant brain region-specific changes in phosphatidylserine and docosahexaenoic acid when compared to wild-type mice [48]. This member of the family has been demonstrated to be directly involved in lipid metabolism by enhancing TAG lipolysis in brown and white adipose tissues, where γ-synuclein was highly expressed [49,50]. In white fat adipocytes isolated from TKO mice, TAG synthesis was further boosted by high fat diet or reduced by caloric restriction [49,50]. 

In this work, we studied how deficiency of all three synucleins affected lipid composition in the brain regions of adult triple knockout (TKO) mice that do not display any signs of pathological changes in the nervous system [51,52]. We suggested that triple ablation would eliminate a well-known effect of functional redundancy and compensatory ability for loss of functions of the absent synuclein family member(s). In addition, in order to get a broader view on possible lipid modulations in transgenic mice, lipids and fatty acid composition of plasma and liver were also investigated. 

## 2. Results

### 2.1. Total Lipids and Lipid Classes in Liver and Plasma from WT and TKO Mice

**Figure 1:**
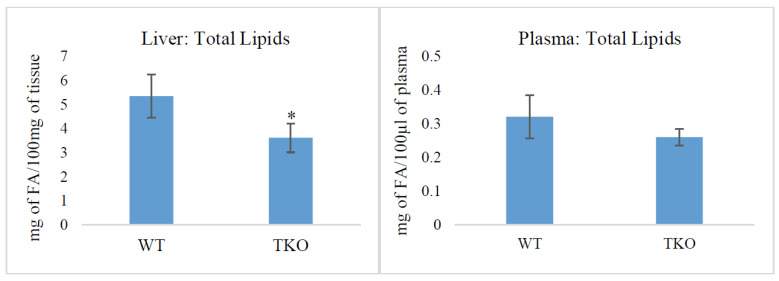
Total lipid content in liver (mg of fatty acids in 100 mg of tissue) and plasma (mg of fatty acids in 100 µL of plasma) from wild-type (WT) and triple-synuclein null mutant (TKO) mice. Means ± s.d. (*n* = 6) are shown. * *p* < 0.05.

Synuclein deficiency resulted in a significant reduction of the total lipids in the liver compared to those in WT animals, whereas the concentrations of total lipids in the plasma were unchanged (Figure 1). In the brain, the total lipid concentrations were unchanged: 2.65 ± 0.78 mg of FA/100 mg of fresh weigh (FW) and 2.17 ± 0.47 mg of FA/100 mg of FW in the midbrain of WT and TKO mice, respectively; 2.06 ± 0.33 mg of FA/100 mg of FW and 2.51 ± 0.53 mg of FA/100 mg of FW in the cortex of WT and TKO mice, respectively. 

**Figure 2:**
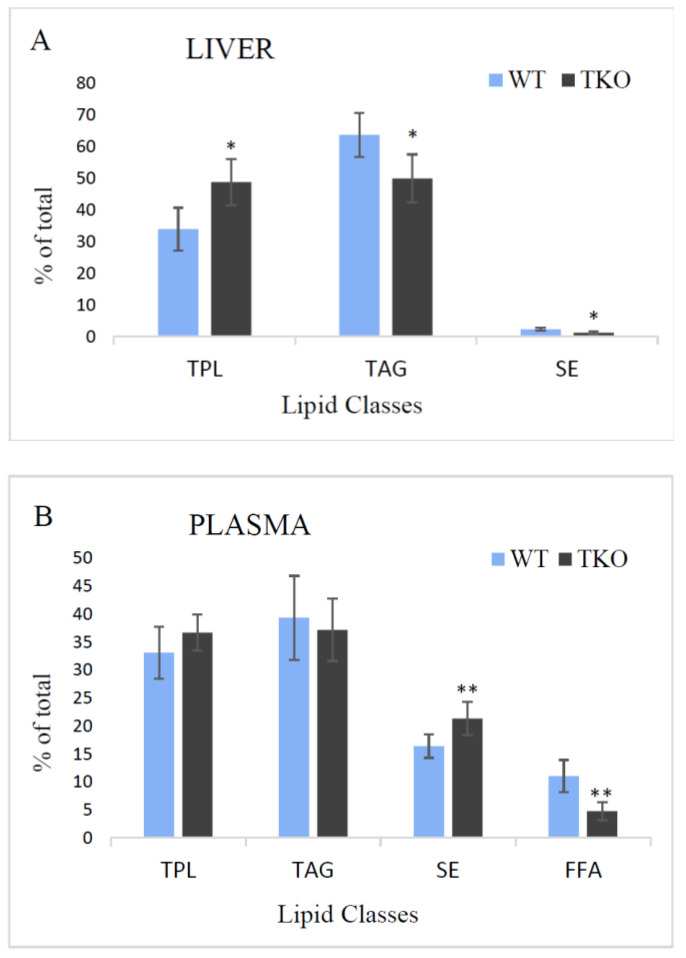
The relative concentrations (% of total) of the major lipid classes in liver (**A**) and plasma (**B**) from wild-type (WT) and triple-synuclein knockout mice: the total polar lipids (TPL), triacylglycerols (TAG), steryl esters (SE), and free (non-esterified) fatty acids (FFA). Values represent means ± s.d. (*n* = 6). * *p* < 0.05, ** *p* < 0.01.

The profiles of lipid classes found in liver and plasma from WT and TKO mice are shown in Figure 2. In the liver, the total polar lipids (TPL) and TAG were the major fractions with SE being a minor class in both, WT and TKO mice. The relative amounts of TPL increased in the liver of TKO animals (from 34 to 48.8%), whereas those of TAG and SE decreased (from 63.7 to 50% and from 2.3 to 1.2%, consequently) (Figure 2A). In the plasma, free fatty acids (FFA, or non-esterified FA) were also determined, and the level of this fraction significantly decreased in TKO mice (4.8%) in comparison to WT animals (11.1%) (Figure 2B). TPL and TAG were unaltered in the plasma of TKO mice; the relative amount of SE was much higher in the plasma than in the liver and increased in TKO mutants up to 21.3% comparing to WT animals (16.4%). 

### 2.2. Fatty Acid Composition of Total Lipids in Liver and Plasma from WT and TKO Mice

Palmitic (C16:0), stearic (C18:0), oleic (C18:1n9), linoleic (C18:2n6, LA), arachidonic (C20:4n6, ARA) and docosahexaenoic (C22:6n3, DHA) acids were the major FA with moderate amounts of dihomo-γ-linolenic (C20:3n6, DGLA), vaccenic (C18:1n7), two isomers of C16:1, palmitoleic (n7) and n9-hexadecenoic acids in plasma and liver of WT and TKO mice (Table S1). The percentages of four major PUFA, namely, LA, ARA, DHA and DGLA, were lower in the plasma of TKO mice compared to WT, whereas the level of palmitic acid was increased in this mouse model. In the liver of TKO animals, the percentage of oleic acid was lower and the percentages of palmitic and DGLA were higher when compared to WT (Table S1). 

### 2.3. Fatty Acid Composition of Lipid Classes in Liver and Plasma from WT and TKO Mice

In liver and plasma, all the separated lipid classes were analysed for their fatty acid composition. FA profiles of TAG isolated from liver and plasma are quite similar with the domination of palmitic and oleic acids followed by LA, stearic and isomers of C16:1. Only minor amounts of PUFA (not-exceeding 2%) were present in plasma and liver with the higher percentage of α-linolenic acid (C18:3n3, ALA) in TAG of the plasma of both, WT and TKO animals (Table S2).

In TAG of plasma and liver, the subtle differences in FA percentages were found when compared WT and TKO mice. In the plasma, only the level of stearic acid was affected by synuclein deficiency: its relative amount was lower in the plasma TAG from TKO mice. In the liver TAG, the percentages of both isomers of C16:1 were higher whereas the level of other monoenoic FA, oleic acid, which is predominant in TAG, was lower and accounted for 52.5% and 47.3% in the liver from WT and TKO mice, respectively (Table S2). 

FA of SE from the plasma are characterised by the high levels of ARA (about 30% in both, WT and TKO mice) and LA accounted for 34% in the plasma SE from WT and for 42.4% in TKO mice (Table S3). The other PUFA, including ALA, DGLA, EPA and DHA were detected in small amounts (about 1–4%) in WT and TKO mice. Saturated palmitic and stearic acids were found in the relative amounts of not-exceeding 6% and 4%, respectively, in WT mice. In TKO mice, the level of palmitic acid was not changed compared to that in WT mice, whereas the relative concentration of oleic acid decreased from 3.7 to 1.9% under synuclein deficiency. In SE fraction isolated from the liver, oleic acid was dominant in both, WT and TKO mice, with a significant decrease in the latter (from 61.1 to 43.2%). Two other major FA in SE of the liver were LA (no differences in its relative amounts were found between WT and TKO mice) and palmitic acid. The percentage of this acid was significantly higher in SE of the liver from TKO animals. The level of other saturated FA, oleic acid, was also increased in TKO mice up to 9.8% in comparison to 4.7% in WT. Additionally, the higher percentages of C16:1n9 and ALA in SE were detected in the liver of TKO mice. No long-chain PUFA, except ARA (1.9% in both WT and mutant mice), were detected in the liver of the studied animals (Table S3).

FA profiles of the TPL fraction isolated from plasma and liver are present in Table 1. In general, FA compositions of this lipid fraction in both samples from WT mice were characterised by the large amounts of saturated FA (palmitic and stearic acids), mono- and dienoic C18 FA (oleic acid and LA) and ARA followed by DHA, DGLA, nervonic acid (C24:1n9), ALA and palmitoleic acid. These parameters were remarkable affected by synuclein deficiency. Thus, in TPL of the plasma from TKO mice, a significant reduction in the level of LA (from 19.2% in WT to 2.7% in TKO) was noted, and only the trace (<0.5%) amounts of ARA, DHA and DGLA were detected. These changes were accompanied by significant increases in the levels of saturated palmitic and monoenic oleic acids (Table 1). In the liver, analysis of FA in TPL fraction also revealed increases in palmitic and oleic acids and a decline in the percentage of ARA. The relative amounts of LA, DGLA and DHA were not altered in TPL fraction in the liver from TKO mice. 

### 2.4. Composition of Free Fatty Acids in Plasma 

**Figure 3:**
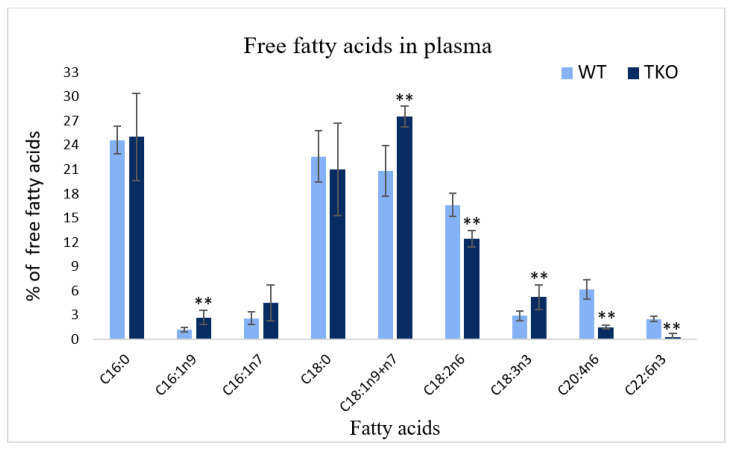
Comparison of free (non-esterified) fatty acids (FFA) in the plasma from wild-type (WT) and triple-synuclein knockout mice (% of total FFA). Values represent means ± s.d. (*n* = 6). ** *p* < 0.01.

A comparison of the profiles of free fatty acids isolated from the plasma of WT and TKO animals is illustrated in Figure 3. In consonance with the data on FA profiles of the total lipids, SE and TPL (described above), the analysis of FFA showed a reduction of the percentages of LA (from 16.6% in WT to 12.4% in TKO), ARA (from 6.2 to 1.5%) and DHA (from 2.5 to 0.3%) together with increases in C16:1n9, oleic acid and ALA. No differences were noted for saturated FA and palmitoleic acid between WT and TKO mice (Figure 3). 

### 2.5. Fatty Acid Composition of Individual Phospholipids in Liver from WT and TKO Mice

**Figure 4:**
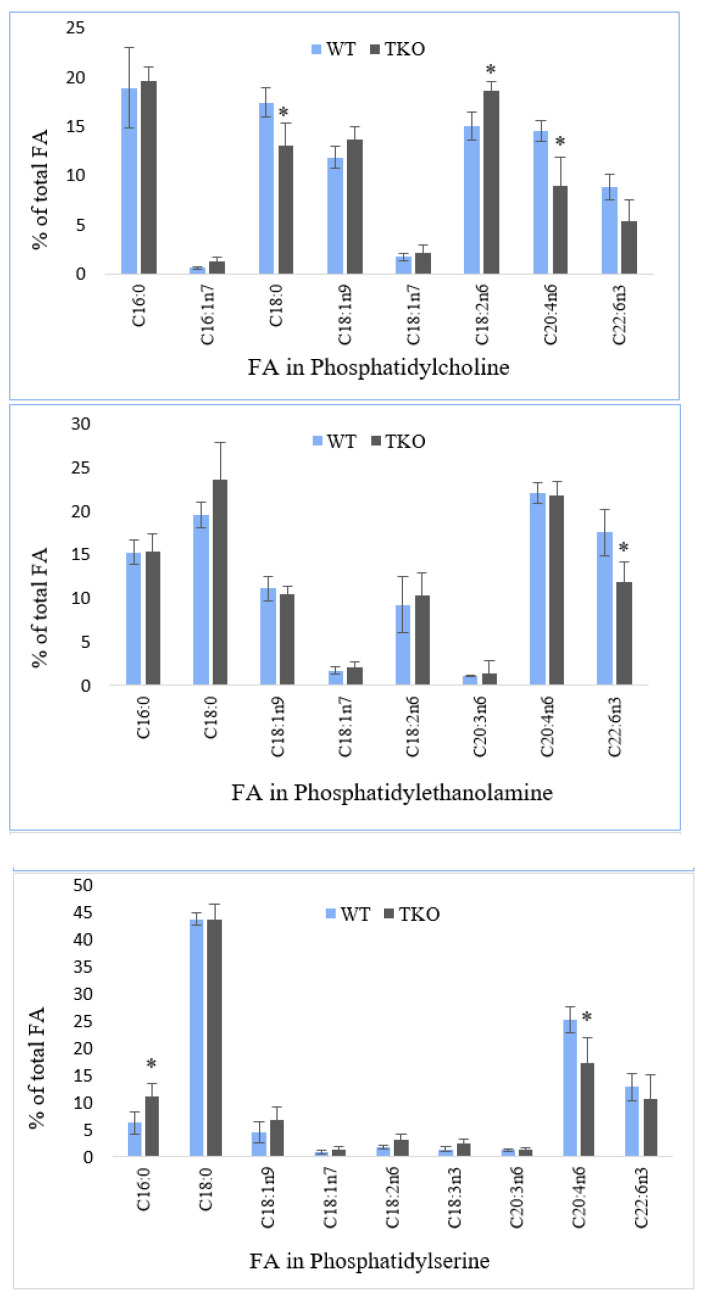

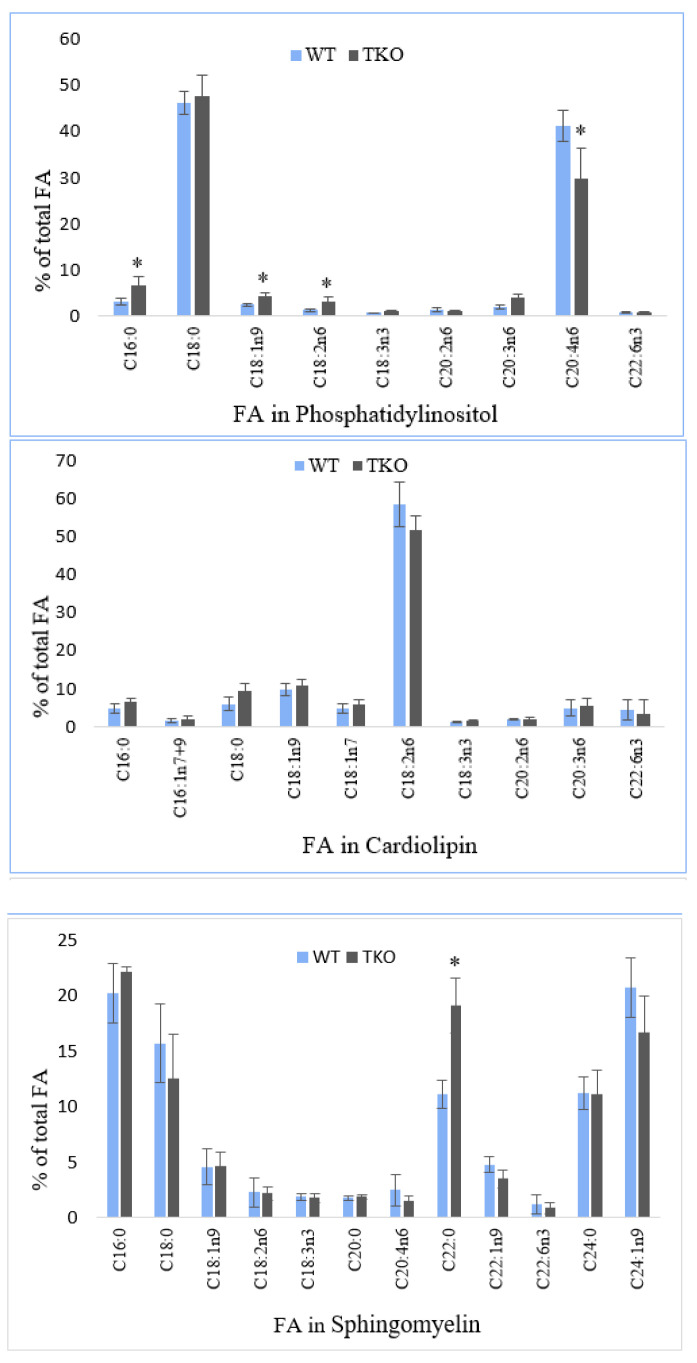
Fatty acid composition in individual polar lipid classes (% of total PL fatty acids) from the liver of wild-type (WT) and triple-synuclein null mutant (TKO) mice. Means ± s.d. (*n* = 6) are shown. Fatty acids are indicated with the number before the colon showing the number of carbon atoms, the figure afterwards denoting the number of double bonds. The position of the first double bond is shown after “n”. Only the major fatty acids (≥0.5%) are present. The asterisk (*) indicates a significant effect of triple-synuclein deficiency when compared with WT (*p* < 0.05).

Figure 4 shows the data on fatty acid composition of individual polar lipids (PL) in the liver from WT and TKO mice. PL composition was in keeping with the generally known lipids found in mammalian livers, particularly: phosphatidylcholine (PC) and phosphatidylethanolamine (PE) as predominant phosphoglycerolipids, followed by phosphatidylserine (PS), phosphatidylinositol (PI), cardiolipin (CL) and sphingomyelin (SM) (data not shown).

The analysis of FA in PC revealed six dominant FA, namely, palmitic, stearic, LA, ARA, oleic and DHA varying in their percentages from 18.9% (palmitate) to 8.8% (DHA) in PC from WT mice. Vaccenic, palmitoleic and ALA were found in low or trace amounts in PC of the liver. In synuclein-free mice, the level of ARA was significantly reduced from 14.5 to 8.9% with a reciprocal increase in the relative amounts of LA (from 15 to 18.6%); the reduced percentage of stearate was also noted in PC of the liver of TKO mice (Figure 4). The liver PE was dominated by the same FA as found in PC, although their relative concentrations were different (Figure 4). Thus, ARA, stearate and DHA showed the highest percentages accounting for 22%, 19.5% and 17.5%, respectively, followed by palmitate (15.2%), oleate (11.1%) and LA (9.2%) in WT mice. Palmitoleic acid was present in trace amounts; vaccenic, eicosapentaenoic (C20:5n3, EPA) and docosapentaenoic (C22:5n3, DPA) acids were the minor FA in PE of the liver from both, WT and TKO mice. Synuclein-deficiency resulted in a decline in DHA percentage and in an appearance of DGLA and gondoic acid in the liver PE in small amounts (0.6–0.8% of the total FA). 

In PS, stearate, ARA and DHA were the dominant acyl chains (43.7%, 25.1% and 12.8%, respectively) with palmitic and oleic acid accounted for 6.2% and 4.5% of the total FA in the liver of WT mice (Figure 4). The minor percentages of vaccenate, LA, ALA, adrenic acid (C22:4n6) and DPA were detected in PS of the liver. A notable decrease in the level of ARA (from 25.1 to 17.3%) alongside with an increase in palmitate (from 6.2 to 11.1%) was found in PS in the liver of TKO mutants. Two FA, stearate and DHA, were prevalent reaching 46.3% and 41.2% in PI of the liver from the studied animals (Figure 4). The moderate amounts of palmitate (3.1%), oleate (2.4%), DGLA (2%), eicosadienoic acid (C20:2n6; 1.4%) and LA (1.3%) as well as the small amounts of vaccenate, ALA and DHA were detected in WT mice. In PI of the liver from TKO mutants, a significant reduction in the percentage of DHA (from 41.2 to 29.9%) was noted. A similar trend was shown for PC and PS (Figure 4). This decline was accompanied by increases in the amounts of palmitate (from 3.1 to 6.6%), oleate (from 2.4 to 4.4%) and LA (from 1.3 to 3.1%) in the liver of synuclein-free mice.

In cardiolipin, LA was accounted for more than 50% of the total fatty acid chains. Other FA with the relative amounts in the range between 4% and 10% were: DHA, DGLA, vaccenate, palmitate, stearate and oleate. The small concentrations of eicosadienoic, arachidic, and trace amounts of ARA and eruric acid (C20:1n9) were also determined. No differences in cardiolipin FA profiles in the liver form WT and TKO mutants were revealed (Figure 4). Sphingomyelin (SM) (sphingophospholipid) was characterised by the appreciable amounts of saturated fatty acids including palmitic (20.2%), stearic (15.7%), behenic (11.1%) and lignoceric (11.2%) acids, as well as a long-chain monoenoic acid, nervonic acid (C24:1n9, 20.7%) in the liver from WT mice. Another shorter chain monoenoic eruric acid and oleic acid were found in the relative amounts of 4.8% and 4.6%, respectively, in WT mice. The small percentages of DHA, ARA, DGLA, the isomers of C16:1 and vaccenic acid were also determined in these samples. Under synuclein deficiency, a significant increase in the level of behenic acid (from 11.1 to 19.1%) was recorded in the liver SM with no statistically significant changes in other acyl chains of this sphingophospholipid (Figure 4). 

### 2.6. Fatty Acid Composition of Total Lipids in Midbrain and Cortex from WT and TKO Mice

The midbrain FA were characterised by the high levels of palmitate and oleate (about 20% of total FA), followed by palmitate (17% in WT and 18% in TKO mice) and DHA (12% and 14% in two groups of mice) (Table S4). The percentages of ARA were 8% in WT animals and 9% in TKO mutants. The levels of adrenic, gondoic and vaccenic acids were in the range of 3–5% of the total FA; small amounts of lignoceric, nervonic, hexacosenoic acid (C26:1n9) and palmitoleic acids were also detected. In this brain region, synuclein deficiency resulted in a slight increase of ARA (from 8.3 ± 0.5% in WT to 9.3 ± 0.4% in TKO mice) in parallel to decreases in the percentages of hexacosenoic (from 2.8 ± 0.2% to 1.8 ± 0.1%), nervonic (from 2.7 ± 0.3% to 1.8 ± 0.2%) and lignoceric (from 0.9 ± 0.1% to 0.6 ± 0.1%) acids (Table S4).

Two major saturated FA, palmitate and stearate, were determined at 21–22% of the total FA in the cortex from WT and TKO animals, followed by DHA (18%), oleate (16%) and ARA (11%). In comparison to FA composition of the midbrain, the lower levels of oleate, vaccenate, gondoic, adrenic, lignoceric, nervonic and hexacosenoic acids alongside with the higher percentages of palmitate, ARA and DHA were found in the cortex (Table S4). No changes in the cortex FA were noted in TKO mice when compared to WT animals. 

### 2.7. Composition of Individual Phospholipids in Midbrain and Cortex from WT and TKO Mice

**Figure 5:**
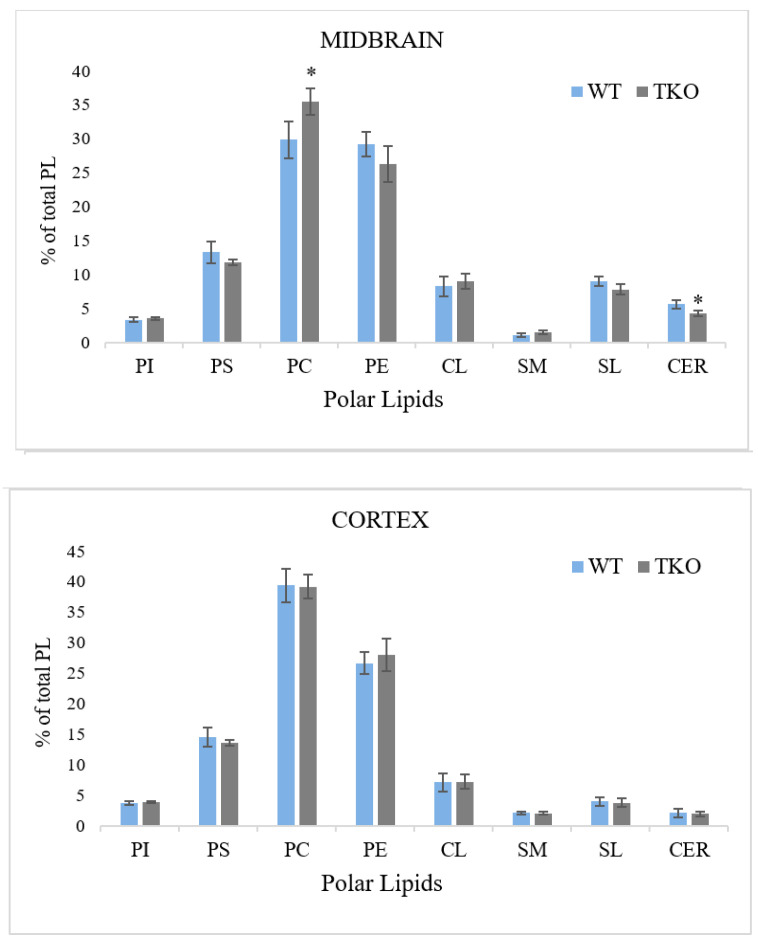
Midbrain and cortex polar lipid composition (% of total polar lipids) from wild-type (WT) and triple-synuclein null mutant (TKO) mice. Means ± s.d. (*n* = 5 for WT cortex; *n* = 6 for WT midbrain and both regions of TKO) are shown. * *p* < 0.05. Abbreviations: PI, phosphatidylinositol; PS, phosphatidylserine; PC, phosphatidylcholine; PE, phosphatidylethanolamine; CL, cardiolipin; SM, sphingomyelin; SL, sulfatide; CER, cerebroside.

Phosphatidylcholine (PC) and phosphatidylethanolamine (PE) were most abundant lipids in both brain regions (around 30–40% of TPL), followed by phosphatidylserine (PS) accounted for about 15% of PL, phosphatidylinositol (PI), sphingomyelin (SM), cardiolipin (CL), sulfatide (SL) and cerebroside (CER): the latter five lipids were found in the percentages not-exceeding 10% of TPL (Figure 5). In the midbrain, the amounts of PC and SM were lower, while the levels of SL, CER and CL were higher than the concentrations of these polar lipids in the cortex. An effect of synuclein ablation was seen only in the midbrain, where the concentration of PC was elevated in TKO mice, and the amount of CER was reduced when compared to WT animals (Figure 5). 

### 2.8. Fatty Acid Composition of Individual Phospholipids in Midbrain and Cortex from WT and TKO Mice

**Figure 6:**
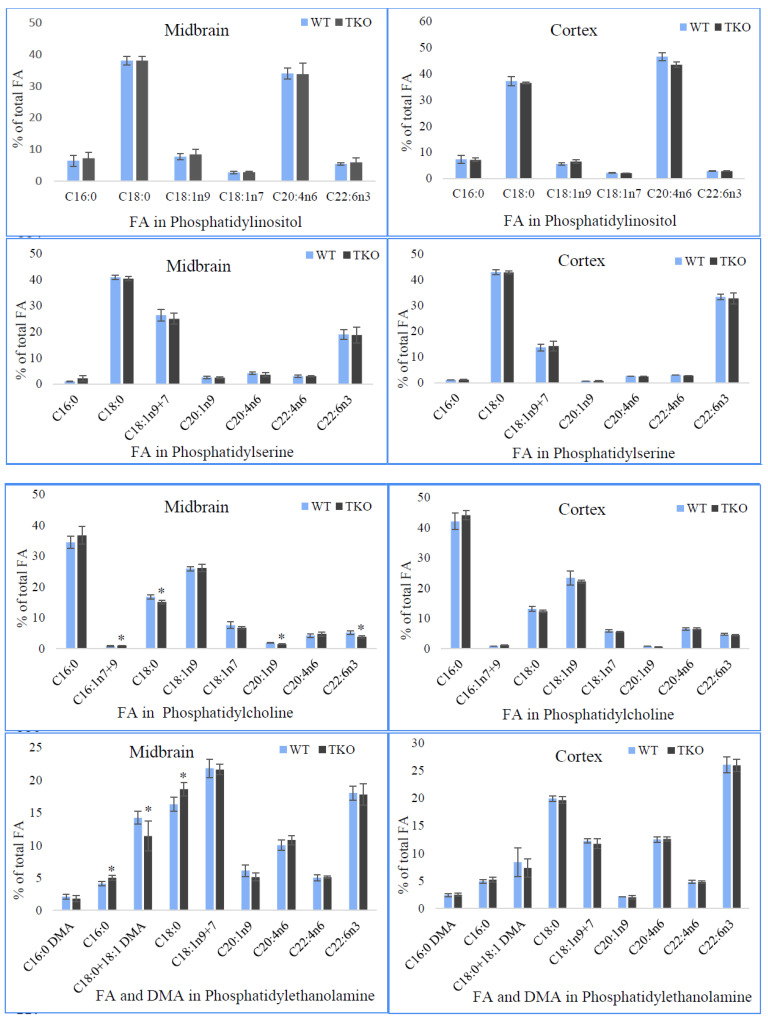

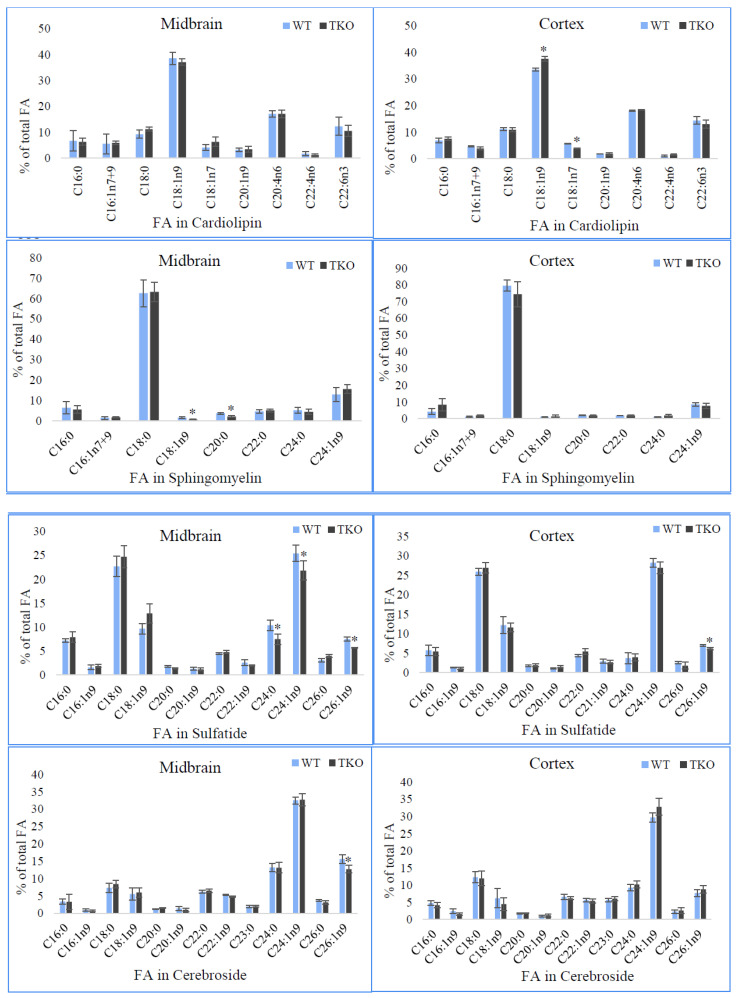
Fatty acid and dimethylacetal (DMA) composition in the individual polar lipid classes (% of total PL fatty acids) from the midbrain and cortex of wild-type (WT) and triple-synuclein null mutant (TKO) mice. For sulfatides and cerebrosides, the conventional (non-hydroxylated) fatty acids are present. Means ± s.d. (*n* = 6) are shown. Fatty acids are indicated with the number before the colon showing the number of carbon atoms, the figure afterwards denoting the number of double bonds. The position of the first double bond is shown after “n”. Only the major fatty acids (≥0.5%) are present. The asterisk (*) indicates a significant effect of triple-synuclein deficiency when compared with WT (*p* < 0.05).

Figure 6 shows the data on fatty acid composition of the individual polar lipids in midbrain and cortex from WT and TKO mice. 

The FA distribution in these lipids is typical of that which has been established in murine brain tissues including our previous study [48]. Analysis of FA in PI showed a domination of stearic acid and ARA in both brain regions from WT and TKO mice with the higher level of ARA in the cortex in comparison to the midbrain (34% vs. 42%) (Figure 6). In the cortex, the lower relative amounts of oleic acid and DHA were also noted. The appreciable amounts of palmitic acid and two isomers of C18:1 acid together with a low (about 0.5% of total FA in PI) level of palmitoleic acid were also determined in PI from both brain regions of the studied mice. In the midbrain, in addition to described FA, gondoic (C20:1n9) and adrenic (C22:4n6) acids were found in the relative amounts of about 1% of FA, whereas in the cortex, these acids were present only in the trace amounts (less than 0.5% of total FA). There were no differences in FA composition in PI in the midbrain and cortex between WT and TKO animals.

In PS, stearic acid, the combined isomers of C18:1 (n7 + n9) and DHA were the major acyl chains. In the cortex of both animal groups, the higher levels of stearate (43% vs. 40–41%) and DHA (33% vs. 19%) and the significantly lower (nearly halved) percentages of C18:1 (n7 + n9) (14% vs. 25–26%) were demonstrated in comparison to the midbrain. In addition, the lower relative amounts of gondoic acid and ARA were found in the cortex. Synuclein-deficiency did not course any changes in FA composition of PS in midbrain and cortex (Figure 6).

Analysis of FA in PC showed the domination of palmitate in the midbrain (34% in WT and 37% in TKO mice) and in the cortex (42% in WT and 44% in TKO mice) of the studied animals. In both brain regions, palmitate and oleate were also determined in the considerable amounts in PC with the slightly lower levels of these acids in the cortex (13% vs. 17%, and 6% vs. 8%, respectively, in WT mice). ARA, DHA and vaccenic acid were present in the range of 4–8% of the total FA in midbrain and cortex of the studied animals. Small amounts of adrenic, gondoic and isomers of C16:1 were determined in this phospholipid from both brain regions. In the midbrain PC of synuclein-free mice, the proportions of DHA, gondoic and stearic were reduced in comparison to WT mice. 

In PE of the midbrain (the other major phospholipid of brain tissue), the dominant FA were C18:1 (22% as two combined isomers), DHA (18%), stearate (16% in WT) and ARA (10–11%). In addition, significant amounts of plasmalogen form of PE, estimated as percentages of dimethyl acetals (DMA), were detected in the proportions of 2.1% of C16:0 DMA and 14% C18:0 + C18:1 DMA in the midbrain with the lower amount of the latter in the cortex (8% in WT mice). PE also contains moderate amounts of palmitic, gondoic, and adrenic acids, as well as a minor quantity of two isomers of C16:1 (Figure 6). A reduction in C18 DMA (from 14.2 ± 1.0% to 11.4 ± 1.3%) in the midbrain as a result of synuclein-deficiency was revealed when comparing PE from WT mice with TKO mutants. This decline was accompanied by a little rise (1–2%) in the relative concentrations of saturated palmitic and stearic acids. In the cortex, FA composition of this lipid was unaffected by synuclein ablation in the studied mouse model (Figure 6). 

Cardiolipin isolated from the brain tissues contains oleate as a dominant FA (39% in the midbrain and 34% in the cortex of WT) followed by ARA, DHA and stearate in the range from 17 to 9% in the midbrain and from 18% and 11% in the cortex of WT mice (Figure 6). Palmitate, isomers of C16:1, vaccenate and gondoic acid were determined in moderate amounts (2–7%) in this lipid from both brain regions. Adrenic acid was present in the relatively small concentrations, 1% and 2% in the midbrain and cortex, respectively. It should be noted that LA, which is a predominant FA in cardiolipin from the liver, was not detected in cardiolipin from the brain samples in this work (as well as in the other analysed polar lipids). These findings are in accordance with the previous studies (see Discussion). Synuclein deficiency affected only FA composition in cardiolipin from the cortex with no changes found in the midbrain. The relative amount of oleic acid was increased in TKO in comparison to WT animals (37.6 ± 0.9% vs. 33.5 ± 0.5%) for the expenses of the level of vaccenic acid. 

The FA profile in SM was characterised by the large amounts of saturated FA, namely, stearate (63% in the midbrain and 80% in the cortex from WT mice) and the moderate percentages of palmitic, lignoceric, behenic and arachidic acids (4–7% in the midbrain from WT mice). The relative concentrations of three latter FA were lower in SM of the cortex from these animals. Monoenoic acids, including nervonic acid and isomers of C16:1 and C18:1 acids, were another group of the acyl chains detected in this lipid with nervonic acid being dominant and counted for 13% in the midbrain and for 9% in the cortex from WT mice. The relative concentration of this acid was much lower (9% vs. 13%) in the cortex, whereas the level of stearic acid was significantly higher (80% vs. 63%) in the cortex when compared to the midbrain from WT mice. In the midbrain of synuclein-free mice, the subtle reductions in the levels of C18:1 isomers (from 1.6 ± 0.4% to 0.7 ± 0.2%) and arachidic acid (from 3.7 ± 0.5% to 2.2 ± 0.4%) were noted. No changes in the FA composition of SM were found in the cortex in TKO mutants.

The composition of the conventional (non-hydroxylated) FA of two major brain glycosphidgolipids, i.e., sulfatides and cerebrosides, is shown in Figure 6. As indicative for these lipid classes, SL and CER contain saturated and monounsaturated (n-9) FA in the range of carbon chains from C16 to C26. Stearic and nervonic acids were the major FA in SL from midbrain and cortex ranging from 22 to 28%. The substantial proportions of oleic, hexacosenoic and palmitic acids were found in this sphingolipid from both brain regions. Some lower but the appreciable amounts of 9-hexadecenoic, arachidic, gondoic, behenic, eruric and lignoceric acids were detected in SL in the brain of the studied animals. In TKO mice, the relative concentrations of lignoceric (7.5 ± 1.1%), nervonic (21.8 ± 2.0%), and hexacosenoic (5.7 ± 0.1%) acids were reduced in comparison to those in WT mice (10.4 ± 1.1%; 25.4 ± 1.7%; 7.5 ± 0.4%, respectively). Analysis of FA in SL of the cortex revealed only a small decline in the proportion of hexacosenoic acid from 7.0 ± 0.2% in WT mice to 6.2 ± 0.3% in synuclein-free mice. 

Three very-long-chain FA, namely, lignoceric (9–13%), nervonic (30–33%) and hexacosenoic (8–16%) acids were predominant in CER isolated from both brain regions of the studied animals (Figure 6). When comparing the FA profiles of this lipid class between midbrain and cortex, the latter was characterised by the higher levels of 9-hexadecenoicstearic, stearic, lignoceric and tricosanoic (C23:0) acids and the lower percentages of hexacosenoic acid. Synuclein-deficiency resulted in the reduced amounts of hexacosenoic acid in CER isolated from the midbrain from 15.6 ± 1.2% in WT to 12.7 ± 1.1% in TKO mice. No changes in CER acyl chains from the cortex between WT and synuclein-free animals were noted in our study (Figure 6). 

## 3. Discussion

Mammalian cells produce a remarkable diversity of fatty acids and lipids, but deeper understanding of molecular complexity of lipids in cells is arising from the technological advances offered by lipidomics [53,54]. Such complexity is the result of many networking processes including the synthesis, trafficking and turnover of lipid compounds which are strongly regulated in different ways in the different cell types and tissues [55]. 

An involvement of synucleins in the modulation of lipid metabolism has been shown by numerous studies [40,41,42,43,44,48]. Special attention has been paid to the complex interactions between lipids (fatty acids and membrane lipids) and synucleins, which have been briefly outlined in the Introduction. Indeed, the previous studies on synuclein—lipid binding have provided pivotal information on the structural properties of these proteins and membrane interactions, but studies of membrane lipids/proteins, using simplified mixtures of the common lipids and in vitro approaches, may not adequately reveal the role of synucleins in membrane binding, reorganisation or signaling. In particular, a link between dysregulation of lipid metabolism and the pathology of Parkinson’s disease has been widely recognised [56,57] and more complex research on the involvement of synucleins in modulation of lipids on the tissue level is needed on the role of synucleins in PD and in the cell in general. Based on this suggestion, we analysed and compared the lipid and fatty acid profiles of plasma, liver and two brain regions in triple-synuclein knockout mice with these parameters in WT animals. 

The liver plays a key role in lipid metabolism and, importantly, a special role in supplying FA to the central nervous system. Lipid metabolism in the liver affects the concentration and composition of secreted lipoproteins (LP) in the plasma and, eventually, the lipids and FA in peripheral tissues.

Our study showed that lipid content in the liver from TKO mice was significantly lower than that in WT animals (Figure 1). This reduction was on the account of the decreased TAG and SE accumulation with no changes (only relative, as %) of TPL (Figure 2). It should be noted that triple synuclein knockout mice show no signs of the dysfunction of their liver or cardiovascular system.

TAG in hepatocytes, depending on the energy demand, are stored in LD and also packed into lipoproteins which are secreted and hydrolysed by lipoprotein lipases in the vascular system. PL synthesis supplies the lipid substrates for VLDL as well as contributes to the biogenesis and the maintenance of ER/Golgi membranes. This has an impact on both, the cargo and the trafficking machinery for VLDL assembly and secretion [58]. PC, cholesteryl esters, FA and TAG affect the VLDL assembly [59]. In our study, no changes in the circulating PL and TAG were found suggesting that synuclein ablation did not affect the amounts of secreted lipids as VLDL compounds in TKO mice despite of the reduced TAG accumulation in the liver, while the FA compositions of circulating lipids were significantly altered. In our opinion, one of the main findings of this work is a remarkable modification of PUFA synthesis and esterification of the major polar lipids by PUFA acyl chains in the liver that was accompanied by an enormous reduction of these FA in the plasma polar lipids. The observed changes will affect the structure/fluidity of surface monolayer of VLDL, their density, the lipases access to TAG and SE during hydrolysis of VLDL and, consequently and more importantly, the supply of PUFA to the peripheral tissues. 

Several mechanisms of synuclein involvement in the modulation of FA and lipid metabolism have been suggested. This protein has structural similarities to the class A2 lipoproteins and some sequence similarity to FABP. In addition, it is present in the large quantities in microsomes, where the synthesis of complex lipids takes place. Since in the brain, but not in the liver, a specific role of α-synuclein in FA uptake and trafficking has been demonstrated, it was hypothesised that α-synuclein may function as FABP in the CNS [60]. For instance, its deletion decreased the palmitic acid and ARA uptake and altered the incorporation of these FA into individual PL classes in mouse brain [60]. 

Some direct evidence for this hypothesis was provided by a study demonstrating that in α-synuclein-deficient astrocytes, the cellular lipid pool has been altered by reducing the uptake of palmitic acid similarly to the effect of FABP3 in cell cultures [61,62]. However, the binding affinity of α-synuclein to PUFA was two orders of magnitude much less than that for FABP; thereby, the role of α-synuclein in modulation of ER-located long chain-acyl-CoA synthetases (ACSL) has been suggested [46,60]. ACSLs are essential in the complex lipid biosynthesis and in FA targeting for incorporation into the specific lipid pools. 

The metabolic fates of various acyl-CoA are determined by a network of proteins that channel them toward or away from the specific pathways to promote FA partitioning [63]. Hepatic FA partitioning requires the interaction of ACSL1 with other specific proteins, such as peroxisomal and lipid droplet proteins, tethering proteins and vesicle proteins that uncovers a dynamic role for ACSL1 in organelle and LD interactions. Proteins involved in lipid metabolism were also identified, including acyl-CoA-binding proteins (ACBP) and ceramide synthase (CerS) isoforms 2 and 5 [64]. Protein complexes composed of several ACSL isoforms have been found in mitochondria. They include ACSL3 and several SNARE proteins, such as SNAP23 (synaptosomal-associated protein 23), VAMP (vesicle-associated membrane protein) and syntaxin 17, suggesting a role for ACSL at membrane contact sites between mitochondria and other organelles or vesicles. In the liver, syntaxin 17 interacts with ACSL3, but not with LD formation-unrelated ACSL1 or ACSL4, through its SNARE domain promoting ACSL3 translocation to the ER to the surface of LD [65]. It should be stressed that more research needed to reveal a role of synuclein deficiency in mitochondrial PUFA synthesis and turnover, since mitochondrial dysfunction is a critical step in the nigral degeneration in PD. 

We suggest that in the absence of all synucleins, ACSL4 activity is reduced impairing the synthesis of PUFA-CoA and their incorporation into hepatic phospholipids, namely, ARA to PC, PS and PI and DHA in PE. As to the possible role of synucleins in TAG storage, VLDL synthesis and secretion in blood, it may be connected to the well-known interactions of synucleins with SNARE proteins (which is in turn also interact with ACSL isoforms) that may modulate the formation and trafficking of TAG/LD and phospholipids to/from the ER/Golgi/mitochondria/peroxisomes. The lipoprotein assembly is regulated by proteins by mediating the interactions with receptors, enzymes and lipid transport proteins. The following components of the SNARE play a role in docking and fusion of the VLDL transport vesicles: Sec22b (vesicle SNARE), syntaxin 5, rBet1 and Gos28 (target membrane SNARE) [66]. Thus, it is tenable to suggest that synucleins can be involved in VLDL synthesis via several anticipated mechanisms. The assembling of VLDL with the trace amounts of PUFA in their polar lipids, that ensured by the trace levels of PUFA in the plasma TPL, implies the mechanisms of specific binding and/or preferential transport of phospholipids esterified mainly with palmitic and palmitoleic acids but not with PUFA to the nascent VLDL. It is noteworthy, that the degree of PUFA esterification of steryl esters in the plasma lipoproteins is substantially higher than that in the liver, and it was not altered by synuclein ablation (Table S3). In additional to the de novo synthesis of SE, the mechanism of SE formation via transferring 2-acyl groups from phospholipids (the *sn*-2 position of glycerol backbones is predominantly esterified by PUFA) to cholesterol also exists. The further (onward the lipoprotein secretion pathway) remarkable reduction of PUFA levels in plasma phospholipids can hereby be explained in TKO mice, where PUFA incorporation into the liver total polar lipids is already impaired. The circulation of highly unsaturated SE in the blood stream of synuclein mutants may ensure a sufficient PUFA supply to peripheral tissues required for the maintaining lipid synthesis and homeostasis despite of the significantly reduced total pool of the circulating PUFA. The increased plasma concentration of SE also contributes to the plasma PUFA level.

Although the plasma lipoproteins are the major transport medium for esterified FA, the appreciable amounts of FA are transported in non-esterified form as bound to albumin. In our study, the total pool of these free FA was significantly reduced in synuclein-ablated animals with a remarkable reduction in the circulating free ARA and DHA (Figure 2 and Figure 3). Passive diffusion across the plasma membrane is well-known to be modulated by FABPs which possess the acyl-CoA synthetase activities that in turn may be regulated by synucleins (as discussed above). Thus, synuclein deficiency markedly affects the hepatic lipid homeostasis as well as the diffusion and transport of PUFA to peripheral tissues. Elucidation of the precise mechanisms of these actions deserves further investigations. In addition, the interaction of α-synuclein with the plasma lipoproteins has been well-documented suggesting an important role the synuclein-related particles in lipid and fatty acid transport in the health conditions and in diseases [67,68]. 

The brain is highly enriched with PUFAs, especially with ARA and DHA, and their uptake from the circulating lipid pool was previously postulated to be essential to maintain a homeostatic pool of LCPUFA in the nervous tissues. Further studies using rodents demonstrated that the synthesis from the essential precursors, namely, LA and ALA, could provide the sufficient ARA and DHA levels for the estimated adult brain requirement for these LCPUFA [69,70]. The synthesis of these LCPUFA from their precursors takes place primarily in the hepatocytes and then transported, as mentioned above, in both, the esterified and non-esterified forms, to the nervous system. 

Besides an uptake of the preformed PUFA from the circulating blood, the appreciable amounts of ARA and DHA can be synthesised from their uptaken precursors (LA, DGLA and ALA) in the microvascular endothelium, oligodendrocytes and astrocytes [71,72]. Neurons themselves are inefficient in the lipid synthesis, although they showed a limited capacity to produce DHA from the omega-3 precursors under the conditions of low dietary supply as well as some elongation of the shorter chain precursors to produce DHA and ARA [71]. 

PUFA play several physiological roles, including the generally known structural role, an importance in the energy storage and production, inflammation and in cell signaling [73]. They regulate the gene expression by affecting the transcription factors, such as hepatic peroxisome proliferator-activated receptor alpha (PPAR-α), sterol regulatory element-binding protein 1 (SREBP-1), carbohydrate-responsive element-binding protein (ChREBP) and max-like protein X (MLX) that regulate proteins which are involved in the lipid synthesis and oxidation, and lipoprotein secretion [74]. At the subcellular level, PUFAs modulate the membrane functioning via influencing lipid rafts, where they alter the raft structural and dynamic characteristics, such as domain size and membrane order, most likely by the lateral segregation of LCPUFA-rich domains from cholesterol-rich membrane regions [73]. The neurophysiological outcomes are substantial because lipid rafts participate in neurotransmission, including the regulation of dopamine transporters, dopamine uptake and dopamine D_2_ receptor oligomerization [75]. 

Polar lipids in the brain are characterised by the very high structural complexity due to a substantial number of the different molecular species of glycerophospholipids and sphingolipids. These two main lipid classes play an important structural and functional role in the cell membranes in general and in lipid rafts. In the midbrain of TKO mice, the levels of C24–C26 sulfatides and cerebrosides were reduced indicating the altered activity of CerS2 that catalyses the acylation of sphingolipids with these long acyl chains. The mechanism of the modulation of CerS2 activity may be connected to the similarity of synucleins to the fatty acid binding proteins or ACSLs, discussed above, since the regulation of very-long acyl chain ceramide synthesis by acyl-CoA binding protein has been demonstrated in mice model work [76].

In the midbrain of TKO mice, a significant increase in PC with a concomitant reduction of cerebrosides was noted a result of synuclein deficiency. Not only the accumulation of these lipid classes was altered, but the remarkable alterations of acyl chains, e.g., decreased DHA in PC and C26:1n9 in ceramides, were also demonstrated. Since the level of DHA was not altered in the midbrain in TKO mutants, we suggest that the channeling of DHA to PC, not its synthesis in the brain, was specifically affected decreasing the unsaturation level of this major brain phospholipid by synuclein deficiency. It is generally accepted, that the changes in membrane fluidity can alter the equilibrium of free versus the membrane bound α-synuclein, and therefore impact the aggregation of α-synuclein into amyloid fibrils. Recently, the importance of the unsaturation level of zwitterionic PC (in addition to previously studied anionic phospholipids) for the conformation and aggregation of N-terminally acetylated α-synuclein has been evidenced [24]. We showed that in the brain, the relative amounts of DHA-enriched PS and its fatty acids were not affected by synuclein deficiency. The importance of this phospholipid in the interaction with N-terminal of α-synuclein has been found to facilitate the SNARE complex formation that was critical for vesicle docking using the single-vesicle docking/fusion assays [77]. Moreover, DHA-enriched PC and PS exhibited the different improvements on 1-methyl-4-phenyl-1,2,3,6-tetrahydropyridine (MPTP)-induced mice with PD by the conducting behavioral experiments: DHA-PS showed the more pronounced effects on PD symptoms and increased the number of dopaminergic neurons in comparison to DHA-PC [78]. As to the additional role of DHA in the brain, its endogenously synthesised endocannabinoid-like metabolite, *N*-docosahexaenoylethanolamine (synaptamide) has emerged as a potent mediator of neurotrophic and neuroprotective effects of DHA [69]. It is interesting that N-arachidonylethanolamine (anandamide) synthesised from ARA, being a structural analogue of synaptamide, possesses the distinctive neuro-bioactivity modulating the brain reward circuitry and neuroinflammation [79]. 

In the liver, PS was enriched with ARA that markedly reduced in TKO mice. This fatty acid is known to stimulate SNARE complex formation and exocytosis, and α-synuclein efficiently blocks ARA-induced SNARE interactions [80]. 

In spite of the remarkable PUFA modifications in the individual lipid classes in liver and plasma of TKO mice, we can conclude that the total supply of PUFA (both, LCPUFA and their short chain essential precursors) was sufficient to maintain the characteristically high level of LCPUFA in midbrain and cortex of the studied mutants. More research on the synthesis of PUFA in the liver and brain cells/tissues is required to understand the mechanisms of its metabolic regulation in the synuclein-free mouse models. 

In the midbrain, a marked reduction in ethanolamine plasmalogens in synuclein free mice can be considered as an important finding, since the abnormal ether lipid levels has been previously implicated in the neurological disorders [81]. Plasmalogens is a class of ether lipids with an ether bond attached to *sn-1* position of the glycerol backbone as opposite to an ester bond of the more common diacyl phospholipids [82]. Plasmalogens are present in mammalian plasma and intracellular membranes. Nervous, immune and cardiovascular systems are enriched in these lipids, where plasmalogens have been proposed to influence membrane dynamics and intracellular signaling being important components of lipid rafts and also act as membrane antioxidants and the reservoir of PUFA [82]. The reduced brain plasmalogen level has been demonstrated in various neurodegenerative and metabolic deregulated diseases which were associated with the increased lipid oxidation. Regarding PD, patients with this disease showed a significant decrease in the post-mortem frontal cortex lipid raft plasmalogens level [82,83]. The results of other studies also showed an importance of plasmalogens for brain functions, albeit a contrary to the hypothesis that the synthesis of these lipids predominantly occurred in the brain and less likely supplied by circulation from peripheral tissues [84]. In our study, the level of plasma DMA among fatty acids of the total polar lipids was non-detectable because of the small sample volumes. Lipidomic approach, which is under our consideration for the future work in this direction, will allow to estimate the level of circulating plasmalogens in TKO mice. From the results obtained in the present study and based on the notion that plasmalogen may function as the potential endogenous antioxidants, we suggest that the reduced level of plasmalogen PE in the midbrain of TKO mice makes the cells more susceptible to the oxidative stress. In support, there is some evidence of involving of plasmalogens in scavenging a variety of reactive oxygen species and in protecting of the unsaturated membrane lipids from oxidation by singlet oxygen [82]. Moreover, the plasmalogen supplementation protected striatal dopamine neurons that degenerated in the response to MPTP-treatment in mice, a PD model [85]. Our results once again emphasise that not only pathological accumulation of synucleins but also their ablation has a profound effect on lipid metabolism. The present study underlined several lipid biosynthetic pathways that were modified by synuclein deficiency in the TKO mouse model. Future research in this direction by studying the highlighted “targets” of synuclein actions as the modulators of lipid synthesis will reveal the mechanisms and the physiological relevance of the crosstalk between synucleins and lipids not only in the brain but at the level of organism in general. Using lipidomic approach for studying lipid rafts (despite of some controversy around the existence of such membrane microdomains [86]), plasmalogen and sphingolipid synthesis, lipoprotein formation and the circulating lipids and fatty acids, as well as for the better understanding of lipid synthesis in astrocytes, will undoubtedly integrate our knowledge of synuclein functions in the cells. 

## 4. Materials and Methods 

### 4.1. Animals 

A line of synuclein-free mice that are null mutants for the all three genes coding for α-, b-, and γ-synuclein was described early [4]. The colony was kept on C57Bl6J (Charles River) background and wild-type control mice on the same C57Bl6J (Charles River) background were bred in the Cardiff University Transgenic Animal Unit. Mice were maintained in conventional open-lid cages with ad libitum access to standard chow and water. Animals were fed a special diet DOI 58Y2 with energy from fats (labDiets). Fatty acid composition of the diet is given in our previous publication [48]. It contained oleic acid as a major compound (35% of total diet FA) followed by linoleic (25%), palmitic (20%), and stearic (13%) acids. The relative amount of a-linolenic (an essential n-3 fatty acid) did not exceed 2.5% of total FA. Lipids accounted for 10% of total energy in this diet. 

Genotyping was carried out by conventional PCR as described previously [4]. Animals (13-month-old male mice) were sacrificed by cervical dislocation and tissues were collected on ice and processed. All animal work was carried out in accordance with the United Kingdom Animals (Scientific Procedures) Act (1986).

### 4.2. Lipid Analysis

The following tissues were used for lipid and fatty acid analysis: plasma, liver, and two brain areas (cortex and midbrain). Lipids were extracted immediately by Folch’s method [87], and lipid extracts were stored at −20 °C under nitrogen atmosphere prior to further analysis. Non-polar lipids were separated using one-dimensional TLC on 10 × 10 cm^2^ silica gel G plates (Merck KGaA, Darmstadt, Germany) in the solvent system hexane/diethyl ether/acetic acid (80:20:1, *v*/*v*/*v*). Polar lipids were separated by two-dimensional TLC on 10 × 10 cm^2^ 1.2% boric acid-impregnated silica gel plates using chloroform/methanol/ammonium hydroxide (65:25:4, *v*/*v*/*v*) in the first dimension and *n*-butanol/acetic acid/water (90:20:20, *v*/*v*/*v*) in the second direction. Lipids were identified by reference to authentic standards and by using specific colour reagent [88]. In addition, several lipid identifications were performed using HPLC-MS-MS (details can be found in our previous publication [48]. Plates were sprayed with 0.2% (*w*/*l*) 8-anilino-4-naphtholenesulphonic acid in dry methanol and viewed under U.V. light to reveal lipids. Individual lipids were scraped from the plates and their fatty acid compositions and contents were determined by gas chromatography (GC) with heptadecanoate (C17:0) as an internal standard. 

### 4.3. Fatty Acid Analysis

Aliquots of the total lipid extracts and individual lipid classes separated by TLC were used for fatty acid methyl esters (FAMEs) preparation. FAMEs were prepared using a solution of 2.5% H_2_SO_4_ in dry methanol/toluene (2:1, *v*/*v*). Samples were heated to 70 °C for 2 h, with occasional vortexing. FAMEs were extracted by two additions of 3 mL of hexane. The hexane fractions were transferred into clean glass tubes, evaporated under a stream of nitrogen, and reconstituted in 50 µL of hexane. All reagents for fatty acid extraction and derivatisation were of HPLC quality.

### 4.4. Gas Chromatography

FAMEs were analysed using a Clarus 500 GC with a flame ionisation detector (Perkin-Elmer, Norwalk, CT, USA). A Perkin-Elmer Elite 225 column (30 m × 0.32 mm × 0.25 µm) was used with a nitrogen carrier gas constant pressure of 20 psi. The oven temperature program began at 170 °C for 3 min, was ramped to 220 °C at 4 °C/min, and was then held for 30 min. FAMEs were identified by comparison of the peak retention times with those of a GC-411 standard mixture (Nu-Chek Prep. Inc., Elysian, MN, USA). Perkin-Elmer TotalChrom software was used for data acquisition and calculation. 

### 4.5. Statistical Analysis

Statistical significance between groups was determined by Mann–Whitney U test. A probability of error less than 5% was considered significant (i.e., *p* < 0.05). The data are reported as mean ± standard deviation of six independent determinations. 

## 5. Conclusions

Our data demonstrate that synuclein deficiency changes the PUFA synthesis and the storage lipid accumulation in the liver. It also results in the reduction of plasmalogens and CER that are the principal compounds of lipid rafts in many tissues. An ablation of all three synuclein family members causes more profound changes in lipid metabolism than changes previously shown to be associated with γ-synuclein deficiency alone.

## Figures and Tables

**Table 1:** Fatty acid composition of the total polar lipids (% of TPL fatty acids) in plasma and liver from triple-synuclein null mutant (TKO) and wild-type (WT) mice.

Fatty Acid	PLASMA		LIVER	
	WT	TKO	WT	TKO
C16:0	23.6 ± 1.2	44.2 ± 5.5 *	21.6 ± 1.5	27.7 ± 3.4 *
C16:1n7	0.7 ± 0.4	1.8 ± 0.6	0.8 ± 0.4	1.2 ± 0.8
C18:0	24.1 ± 1.0	28.4 ± 4.7	22.4 ± 1.9	21.2 ± 2.8
C18:1n9	12.7 ± 0.9	18.1 ± 1.0 *	13.0 ± 1.0	15.2 ± 0.8 *
C18:2n6	19.2 ± 1.4	2.7 ± 0.8 *	13.5 ± 1.1	14.4 ± 0.7
C18:3n3	0.9 ± 0.2	0.8 ± 0.1	tr.	tr.
C20:3n6	1.6 ± 0.2	tr.	1.7 ± 0.3	1.8 ± 0.3
C20:4n6	10.0 ± 1.3	tr.	15.7 ± 1.7	10.4 ± 2.7 *
C22:6n3	4.0 ± 0.6	tr.	7.6 ± 1.4	4.2 ± 2.2
C24:1n9	1.4 ± 0.2	1.3 ± 0.4	0.9 ± 0.1	0.7 ± 0.1

Data as means ± s.d. (*n* = 6). Fatty acids are indicated with the number before the colon showing the number of carbon atoms, the figure afterwards denoting the number of double bonds. The position of the first double bond is shown after “n”. Only the major fatty acids (≥0.5%) are listed; tr., trace, <0.5%. The asterisk (*) indicates a significant effect of triple-synuclein deficiency when compared with WT (*p* < 0.05).

## Supplementary Materials

The following are available online, Table S1: Fatty acid composition of total lipids (% of total fatty acids) in plasma and liver from triple-synuclein null mu-tant (TKO) and wild-type (WT) mice, Table S2: Fatty acid composition of triacylglycerols (% of TAG fatty acids) in plasma and liver from triple-synuclein null mutant (TKO) and wild-type (WT) mice, Table S3: Fatty acid composition of steryl esters (% of SE fatty acids) in plasma and liver from triple-synuclein null mu-tant (TKO) and wild-type (WT) mice, Table S4: Fatty acid composition of total lipids (% of total fatty acids) in midbrain and cortex from triple-synuclein null mutant (TKO) and wild-type (WT) mice.

## Abbreviations

ACSL	long chain-acyl-CoA synthetases
ALA	α-linolenic acid
ARA	arachidonic acid
ER	cerebrosides
ChREBP	carbohydrate-responsive element-binding protein
CL	cardiolipin
DGLA	dihomo-γ-linolenic acid
DHA	docosahexaenoic acid
DMA	dimethyl acetals
EPA	eicosapentaenoic acid
FA	fatty acids
FABS	fatty acid binding protein
FAME	fatty acid methyl esters
GC	gas chromatography
HPLC-MS-MS	high-performance liquid chromatography-mass spectrometry
LA	linoleic acid
LCPUFA	long-chain polyunsaturated fatty acids
LP	lipoproteins
MLX	max-like protein X
MPTP	1-methyl-4-phenyl-1,2,3,6-tetrahydropyridine
NAC	non-Aβ component
PC	phosphatidylcholine
PD	Parkinson’s disease
PE	phosphatidylethanolamine
PI	phosphatidylinositol
PPAR-α	peroxisome proliferator-activated receptor alpha
PS	phosphatidylserine
SE	steryl esters
SL	sulfatide
SM	sphingomyelin
SNAP23	synaptosomal-associated protein 23
SNARE	*N*- ethylmaleimide-sensitive factor attachment protein receptors
SREBP-1	sterol regulatory element-binding protein 1
SV	synaptic vesicle
TAG	triacylglycerol
TKO	triple synuclein-knockout
TPL	total polar lipids
VLDL	very low-density lipoproteins
WT+13:1311:15:39	wild type

## References

[1] George J.M. (2001). The synucleins. Gen. Biol..

[2] Bendor J., Logan T., Edwards R.H. (2013). The functions of α-synuclein. Neuron.

[3] Yuan J., Zhao Y. (2013). Evolutionary aspects of the synuclein super-family and sub-families based on large-scale phylogenetic and group-discrimination analysis. Biochem. Biophys. Res. Commun..

[4] Connor-Robson N., Peters O., Millership S., Ninkina N., Buchman V.L. (2016). Combinational losses of synucleins reveal their differential requirements for compensating age-dependent alterations in motor behaviour and dopamine metabolism. Neurobiol. Aging.

[5] Longhena F., Faustini G., Spillantini M.G., Bellucci A. (2019). Living in promiscuity: The multiple partners of alpha-synuclein at the synapse in physiology and pathology. Int. J. Mol. Sci..

[6] Maroteaux L., Campanelli J.T., Scheller R.H. (1988). Synuclein: A neuron-specific protein localized to the nucleus and presynaptic nerve terminal. J. Neurosci..

[7] Brown J.W.P., Buell A.K., Michaels T.C.T., Meisl G., Carozza J., Flagmeier P., Vendruscolo M., Knowles T.P.J., Dobson C.M., Galvagnion C. (2016). β-Synuclein suppresses both the initiation and amplification steps of α-synuclein aggregation via competitive binding to surfaces. Sci. Rep..

[8] Fusco G., Sanz-Hernandes M., De Simone A. (2018). Order and disorder in the physiological membrane binding of α-synuclein. Curr. Opin. Struct. Biol..

[9] Ohtake H., Limprasert P., Fan Y., Onodera O., Kakita A., Takahashi H., Bonner L.T., Tsuang D.W., Murray I.V., Lee V.M. (2004). Beta-synuclein gene alterations in dementia with Lewy bodies. Neurology.

[10] Ducas V.C., Rhoades E. (2012). Quantifying interactions of β-synuclein and γ-synuclein with model membranes. J. Mol. Biol..

[11] Raina A., Leite K., Chakrabarti K.S., Guerin S., Mahajani S.U., Voll D., Becker S., Griesinger C., Bähr M., Kügler S. (2021). Dopamine promotes the neurodegenerative potential of β-synuclein. J. Neurochem..

[12] Biere A.L., Wood S.J., Wypych J., Steavenson S., Jiang Y., Anafi D., Jacobsen F.W., Jarosinski M.A., Wu G.M., Louis J.C. (2000). Parkinson’s disease-associated alpha-synuclein is more fibrillogenic than beta- and gamma-synuclein and cannot cross-seed its homologs. J. Biol. Chem..

[13] Sung Y.-H., Eliezer D. (2006). Secondary structure and dynamics of micelle bound β- and γ-synuclein. Protein Sci..

[14] Mochizuki H., Choong C.-J., Masliah E. (2018). A refined concept: α-synuclein dysregulation disease. Neurochem. Int..

[15] Roberts H.L., Brown D.R. (2015). Seeking a mechanism for the toxicity and oligomeric α-synuclein. Biomolecules.

[16] Burré J., Sharma M., Südhof T.C. (2012). Systematic mutagenesis of α-synuclein reveals distinct sequence requirements for physiological and pathological activities. J. Neurosci..

[17] Burré J., Sharma M., Tsetsenis T., Buchman V., Etherton M.R., Südhof T.C. (2010). Alpha-synuclein promotes SNARE-complex assembly in vivo and in vitro. Science.

[18] Stefanis L. (2012). a-Synuclein in Parkinson’s disease. Cold Spring Harb. Perspect. Med..

[19] Iyer A., Glaessens M.A.E. (2018). Disruptive membrane interactions of alpha-synuclein aggregates. Biochem. Biophys. Acta Proteins Proteom..

[20] Ho G.H.H., Ramalingam N., Imberdis T., Wilkie E.C., Dettmer U., Selkoe D.J. (2021). Upregulation of cellular palmitoylation mitigates α-synuclein accumulation and neurotoxicity. Mov. Disord..

[21] Kaur U., Lee J.C. (2020). Unroofing site-specific α-synuclein-lipid interactions at the plasma membrane. Proc. Natl. Acad. Sci. USA.

[22] Middleton E.R., Rhoades E. (2010). Effects of curvature and composition of α-synuclein binding to lipid vesicles. Biophys. J..

[23] Lee H.J., Cho E.D., Lee K.W., Kim J.H., Cho S.G., Lee S.J. (2013). Autophagic failure promotes the exocytosis and intercellular transfer of α-synuclein. Exp. Mol. Med..

[24] O’Leary E.I., Jiang Z., Strub M.P., Lee J.C. (2018). Effects of phosphatidylcholine membrane fluidity on the conformation and aggregation of N-terminally acetylated α-synuclein. J. Biol. Chem..

[25] Fusco G., De Simone A., Tata G., Vostrikov V., Vendruscolo M., Dobson C.M., Veglia G. (2014). Direct observation of the three regions in α-synuclein that determine its membrane-bound behaviour. Nat. Commun..

[26] Jao C.C., Der-Sarkissian A., Chen J., Langen R. (2004). Structure of membrane-bound alpha-synuclein studied by site-directed spin labeling. Proc. Natl. Acad. Sci. USA.

[27] Jao C.C., Hegde B.G., Chen J., Haworth I.S., Langen R. (2008). Structure of membrane-bound α-synuclein from site-directed spin labelling and computational refinement. Proc. Natl. Acad. Sci. USA.

[28] Wietek J., Haralampiev I., Amoussouvi A., Herrmann A., Stöckl M. (2013). Membrane bound α-synuclein in fully embedded in the lipid bilayer while segments with higher flexibility remain. FEBS Lett..

[29] Westphal C.H., Chandra S.S. (2013). Monomeric synucleins generate membrane curvature. J. Biol. Chem..

[30] Varkey J., Isas J.M., Mizuno N., Jensen M.B., Bhatia V.K., Jao C.C., Petrlova J., Voss J.C., Stamou D.G., Steven A.C. (2010). Membrane curvature induction and tabulation are common features of synucleins and apolipoproteins. J. Biol. Chem..

[31] Ouberai M.M., Wang J., Swann M.J., Galvagnion C., Guilliam T., Dobson C.M., Welland M.E. (2013). α-Synuclein senses lipid packing defects and induces lateral expansion of lipids leading to membrane remodelling. J. Biol. Chem..

[32] Braun A.R., Lacy M.M., Ducas V.C., Rhoades E., Sachs J.N. (2017). α-Synuclein’s uniquely long amphipathic helix enhances its membrane binding and remodeling capacity. J. Membr. Biol..

[33] Burré J., Sharma M., Südhof T.C. (2014). Alpha-synuclein assembles into higher-order multimers upon membrane binding to promote SNARE complex formation. Proc. Natl. Acad. Sci. USA.

[34] Hannestag J.K., Rocha S., Agnarsson B., Zhdanov V.P., Wittung-Strafshede P., Höök F. (2020). Single-vesicle imaging reveals lipid-selective and stepwise membrane disruption by monomeric α-synuclein. Proc. Natl. Acad. Sci. USA.

[35] Fusco G., Chen S.W., Williamson P.T.F., Cascella R., Perni M., Jarvis J.A., Cecchi C., Vendruscolo M., Chiti F., Cremades N. (2017). Structure basis of membrane disruption and cellular toxicity by α-synuclein oligomers. Science.

[36] Bousquet M., Gue K., Emond V., Julien P., Kang J.X., Cicchetti F., Calon F. (2011). Transgenic conversion of omega-6 into omega-3 fatty acids in a mouse model of Parkinson’s disease. J. Lipid Res..

[37] Galvagnion C. (2017). The role of lipids interacting with α-synuclein in the pathogenesis of Parkinson’s disease. J. Parkinsons Dis..

[38] De Franceschi G., Fecchio C., Sharon R., Schapira A.H.V., Proukakis C., Bellotti V., De Laureto P.P. (2017). α-Synuclein structural features inhibit harmful polyunsaturated fatty acid oxidation, suggesting roles in neuroprotection. J. Biol. Chem..

[39] Suzuki M., Sango K., Wada K., Nagai Y. (2018). Pathological role of lipid interaction with α-synuclein in Parkinson’s disease. Neurochem. Int..

[40] Alza N.P., Iglesias P.A., Conde M.A., Uranga R.M., Salvador G.A. (2019). Lipids at the crossroad of α-synuclein function and dysfunction; biological and pathological implications. Front. Cell Neurosci..

[41] Kiechle V., Grozdanov V., Danzer K.M. (2020). The role of lipids in the initiation of α-synuclein misfolding. Front. Cell Dev. Biol..

[42] Fanning S., Selkoe D., Dettmer U. (2021). Vesicle trafficking and lipid metabolism in synucleonopathy. Acta Neuropath..

[43] Mori A., Hattori N. (2020). Lipids: Key players that modulate α-synuclein toxicity and neurodegeneration in Parkinson’s disease. Int. J. Mol. Sci..

[44] Ugalde C.L., Lawson V.A., Finkelstein D.I., Hill A.F. (2019). The role of lipids in α-synuclein misfolding and neurotoxicity. J. Biol. Chem..

[45] Sánchez Campos S., Alza N.P., Salvador G.A. (2018). Lipid metabolism alterations in the neuronal response to A53T α-synuclein and Fe-induced injury. Arch. Biochem. Biophys..

[46] Golovko M.Y., Faergeman N.J., Cole N.B., Castagnet P.I., Nussbaum R.L., Murphy E.R. (2005). Alpha-synuclein gene deletion decreases brain palmitate uptake and alters the palmitate metabolism in the absence of alpha-synuclein palmitate binding. Biochemistry.

[47] Golovko M.Y., Rosenberger T.A., Feddersen S., Faergeman N.J., Murphy E.J. (2007). Alpha-synuclein gene ablation increases docosahexaenoic acid incorporation and turnover in brain phospholipids. J. Neurochem..

[48] Guschina I., Millership S., O’Donnell V., Ninkina N., Harwood J., Buchman V.L. (2011). Lipid classes and fatty acid patterns are altered in the brain of γ-synuclein null mutant mice. Lipids.

[49] Millership S.J., Ninkina N., Guschina I., Norton J., Brambilla R., Oort P., Adams S., Dennis R.J., Voshol P., Rochford J. (2012). Increased lipolysis and altered lipid homeostasis protect γ-synuclein null mutant mice from diet-induced obesity. Proc. Natl. Acad. Sci. USA.

[50] Millership S., Ninkina N., Rochford J.J., Buchman V.L. (2013). Gamma-synuclein is a novel player in the control of body lipid metabolism. Adipocyte.

[51] Greten-Harrison B., Polydoro M., Morimoto-Tomita M., Diao L., Williams A.M., Nie E.H., Makani S., Tian N., Castillo P.E., Buchman V.L. (2010). αβɣ-Synuclein triple knockout mice reveal age-dependent neuronal dysfunction. Proc. Natl. Acad. Sci. USA.

[52] Anwar S., Peters O., Millership S., Ninkina N., Doig N., Connor-Robson N., Threlfell S., Kooner G., Deacon R.M., Bannerman D.M. (2011). Functional alterations to the nigrostriatal system in mice lacking all three members of the synuclein family. J. Neurosci..

[53] Sethi S., Brietzke E. (2017). Recent advances in lipidomics: Analytical and clinical perspectives. Prostag. Oth. Lipid Med..

[54] Gross R.W. (2017). The evolution of lipidomics through space and time. Biochim. Biophys. Acta Mol. Cell Biol. Lipids.

[55] Aureli M., Grassi S., Prioni S., Sonnino S., Prinetti A. (2015). Lipid membrane domain in the brain. Biochim. Biophys. Acta.

[56] Selkoe D., Dettmer U., Luth E., Kim N., Newman A. (2014). Defining the native state of molecular aspects of medicine. Neurodegener. Dis..

[57] Alecu I., Bennett S.A.L. (2019). Dysregulated lipid metabolism and its role in α-synucleinopathy in Parkinson’s disease. Front. Neurosci..

[58] Sundaram M., Yao Z. (2010). Recent progress in understanding protein and lipid factors affecting hepatic VLDL assembly and secretion. Nutr. Metab..

[59] Mason T.M. (1998). The role of factors that regulate the synthesis and secretion of very-low-density lipoprotein by hepatocytes. Crit. Rev. Clin. Lab. Sci..

[60] Golovko M.Y., Barceló-Coblijn G., Castagnet P.I., Austin S., Combs C.K., Murphy E.J. (2009). The role of α-synuclein in brain lipid metabolism: A downstream impact on brain inflammatory response. Mol. Cell. Biol..

[61] Sharon R., Bar-Joseph I., Mirick G.E., Serhan C.N., Selkoe D.J. (2003). Altered fatty acid composition of dopaminergic neurons expressing alpha-synuclein and human brains with alpha-synucleinopathies. J. Biol. Chem..

[62] Sepe F.N., Chiasserini D., Parnetti L. (2018). Role of FABP3 as biomarker in Alzheimer’s disease and synucleinopathies. Future Neurol..

[63] Grevengoed T.J., Klett E.L., Coleman R.A. (2014). Acyl-CoA metabolism and partitioning. Annu. Rev. Nutr..

[64] Young P.A., Senkal C.E., Suchanek A.L., Grevengoed T.J., Lin D.D., Zhao L., Crunk A.E., Klett E.L., Füllekrug J., Obeid L.M. (2018). Long-chain acyl-CoA synthetase 1 interacts with key proteins that activate and direct fatty acids into niche hepatic pathways. J. Biol. Chem..

[65] Kimura H., Arasaki K., Ohsaki Y., Fujimoto T., Ohtomo T., Yamada J.M., Tagaya M. (2018). Syntaxin 17 promotes lipid droplet formation by regulating the distribution of acyl-CoA synthetase 3. J. Lipid Res..

[66] Ramasamy I. (2014). Recent advances in physiological lipoprotein metabolism. Clin. Chem. Lab. Med..

[67] Emamzadeh F.N., Allsop D. (2017). α-Synuclein interactions with lipoproteins in plasma. J. Mol. Neurosci..

[68] Surguchov A. (2017). Commentary: α-synuclein interactions with lipoproteins in plasma. Front. Mol. Neurosci..

[69] Kim H.Y., Spector A.A. (2018). *N*-Docosahexaenoylethanolamine: A neurotrophic and neuroprotective metabolite of docosahexaenoic acid. Mol. Asp. Med..

[70] Lacombe R.J.S., Chouinard-Watkins R., Bazinet R.P. (2018). Brain docosahexaenoic acid uptake and metabolism. Mol. Asp. Med..

[71] Moore S.A., Yoder E., Murphy S., Dutton G.R., Spector A.A. (1991). Astrocytes, not neurons, produce docosahexaenoic acid (22:6 omega-3) and arachidonic acid (20:4 omega-6). J. Neurochem..

[72] Hofmann K., Rodriguez-Rodriguez R., Gaebler A., Casals N., Scheller A., Kuerschner L. (2017). Astrocytes and oligodendrocytes in grey and white matter regions of the brain metabolize fatty acids. Sci. Rep..

[73] Liu J.J., Green P., Mann J.J., Rapoport S.I., Sublette M.E. (2015). Pathways of polyunsaturated fatty acid utilization: Implications for brain function in neuropsychiatric health and disease. Brain Res..

[74] Jump D.B. (2008). N-3 polyunsaturated fatty acid regulation of hepatic gene transcription. Curr. Opin. Lipidol..

[75] Celver J., Scharma M., Kovoor A. (2012). D (2)-Dopamine receptors target regulator of G protein signaling 9–2 to detergent-resistant membrane fractions. J. Neurochem..

[76] Ferreira N.S., Engelsby H., Neess D., Kelly S.L., Volpert G., Merrill A.H., Futerman A.H., Færgeman N.J. (2017). Regulation of very-long acyl chain ceramide synthesis by acyl-CoA-binding protein. J. Biol. Chem..

[77] Lou X., Kim J., Hawk B.J., Shin Y.K. (2017). α-Synuclein may cross-bridge v-SNARE and acidic phospholipids to facilitate SNARE-dependent vesicle docking. Biochem. J..

[78] Wang C., Wang D., Xu J., Yanagita T., Xue C., Zhang T. (2018). DHA enriched phospholipids with different polar groups (PC and PS) had different improvements on MPTP-induced mice with Parkinson’s disease. J. Funct. Foods.

[79] Scherma M., Masia P., Satta V., Fratta W., Fadda P., Tanda G. (2019). Brain activity of anandamide: A rewarding bliss?. Acta Pharmacol. Sin..

[80] Darios F., Ruipérez V., López I., Villanueva J., Gutierrez L.M., Davletov B. (2010). Alpha-synuclein sequesters arachidonic acid to modulate SNARE-mediated exocytosis. EMBO Rep..

[81] Dorninger F., Forss-Petter S., Berger J. (2017). From peroxisomal disorders to common neurodegenerative diseases–the role of ether lipids in the nervous system. FEBS Lett.

[82] Dean J.M., Lodhi I.J. (2018). Structural and functional roles of ether lipids. Protein Cell.

[83] Fabelo N., Martín V., Santpere G., Marín R., Torrent L., Ferrer I., Díaz M. (2011). Severe alterations in lipid composition of frontal cortex lipid rafts from Parkinson’s disease and incidental Parkinson’s disease. Mol. Med..

[84] Honsho M., Fujiki Y. (2017). Plasmalogen homeostasis - regulation of plasmalogen biosynthesis and its physiological consequence in mammals. FEBS Lett..

[85] Miville-Godbout E., Bourque M., Morissette M., Al-Sweidi S., Smith T., Mochizuki A., Senanayake V., Jayasinghe D., Wang L., Goodenowe D. (2016). Plasmalogen augmentation reserves striatal dopamine loss in MPTP mice. PLoS ONE.

[86] Levental I., Levental K., Heberle F. (2020). Lipid rafts: Controversies resolved, mysteries remain. Trends Cell Biol..

[87] Folch J., Lees M., Stanley G.H.S. (1957). A simple method for the isolation and purification of total lipids from animal tissues. J. Biol. Chem..

[88] Kates M. (1986). Techniques of Lipidology: Isolation, Analysis and Identification of Lipids.

