# Supplementary material for: Triple-Knockout, Synuclein-Free Mice Display Compromised Lipid Pattern
Source: Molecules. 2021 May 21;26(11):3078. doi: 10.3390/molecules26113078 (PMC8196748; doi:10.3390/molecules26113078)
Supplement: Supplementary file 1 [file molecules-26-03078-s001.zip › molecules-1193807-supplementary.pdf]

molecules

MDPI

Article

Triple-Knockout, Synuclein-Free Mice Display Compromised Lipid Pattern

Irina A. Guschina [1,*,†], Natalia Ninkina [1,2,†], Andrei Roman [1,2], Mikhail V. Pokrovskiy [3] and Vladimir L. Buchman [1,2]

Table S1 Fatty acid composition of total lipids (% of total fatty acids) in plasma and liver from triple-synuclein null mutant (TKO) and wild-type (WT) mice

	PLASMA		LIVER	
Fatty acid	WT	TKO	WT	TKO
C16:0	17.4 ± 1.0	24.1 ± 2.7*	19.2 ± 0.6	22.3 ± 2.2*
C16:1n9	0.5 ± 0.1	0.7 ± 0.0 *	0.9 ± 0.2	1.0 ± 0.1
C16:1n7	1.9 ± 0.4	2.2 ± 1.2	1.6 ± 0.5	2.1 ± 0.8
C18:0	12.0 ± 1.5	11.3 ± 1.7	8.5 ± 0.9	9.3 ± 1.5
C18:1n9	24.0 ± 1.8	24.3 ± 2.9	33.1 ± 4.0	24.1 ± 2.2*
C18:1n7	1.3 ± 0.2	1.7 ± 0.2*	1.8 ± 0.4	1.8 ± 0.5
C18:2n6	21.8 ± 1.8	21.4 ± 1.6	16.7 ± 2.0	16.4 ± 0.7
C18:3n3	0.8 ± 0.5	1.1 ± 0.1	0.5 ± 0.1	0.5 ± 0.1
C20:3n6	1.1 ± 0.1	0.6 ± 0.1*	1.2 ± 0.2	1.8 ± 0.1*
C20:4n6	11.7 ± 2.0	7.1 ± 0.8*	8.3 ± 1.2	10.2 ± 1.6
C20:5n3	0.7 ± 0.1	0.9 ± 0.2	tr.	tr.
C22:6n3	4.8 ± 0.8	2.3 ± 0.1*	5.7 ± 1.1	6.8 ± 0.7

Data as means ± SD (n = 6). Fatty acids are indicated with the number before colon showing the number of carbon atoms, the figure afterwards denoting the number of double bonds. The position of the first double bond is shown after "n". Only the major fatty acids (≥ 0.5%) are listed; tr., trace < 0.5%. The asterisk (*) indicates a significant effect of triple-synuclein deficiency when compared with WT ($p < 0.05$).

Table S2 Fatty acid composition of triacylglycerols (% of TAG fatty acids) in plasma and liver from triple-synuclein null mutant (TKO) and wild-type (WT) mice

	PLASMA		LIVER	
Fatty acid	WT	TKO	WT	TKO
C16:0	19.6 ± 1.4	18.9 ± 4.1	17.7 ± 1.4	20.3 ± 2.6
C16:1n9	0.9 ± 0.1	1.1 ± 0.5	1.5 ± 0.3	2.3 ± 0.2*
C16:1n7	3.7 ± 1.1	4.3 ± 1.9	1.9 ± 1.0	3.7 ± 1.3*
C18:0	6.6 ± 1.6	3.9 ± 0.9*	2.2 ± 0.3	1.8 ± 0.3
C18:1n9	45.4 ± 1.0	44.5 ± 6.1	52.5 ± 3.5	47.3 ± 2.2*
C18:2n6	18.1 ± 1.2	20.3 ± 1.6	18.9 ± 2.8	17.9 ± 1.8
C18:3n3	1.5 ± 0.3	1.4 ± 0.3	0.6 ± 0.1	0.8 ± 0.2
C20:4n6	1.0 ± 0.3	1.2 ± 0.2	1.0 ± 0.3	1.1 ± 0.3
C22:6n3	1.5 ± 0.4	1.8 ± 0.3	1.5 ± 0.7	1.6 ± 0.4

Data as means ± SD (n = 6). See legend to Table 1S for other details. The asterisk (*) indicates a significant effect of triple-synuclein deficiency when compared with WT ($p < 0.05$).

Table S3 Fatty acid composition of steryl esters (% of SE fatty acids) in plasma and liver from triple-synuclein null mutant (TKO) and wild-type (WT) mice

	PLASMA		LIVER	
Fatty acid	WT	TKO	WT	TKO
C16:0	5.6 ± 0.9	4.5 ± 0.9	11.8 ± 1.3	18.6 ± 2.4*
C16:1n9	1.1 ± 0.4	1.0 ± 0.1	1.3 ± 0.2	2.8 ± 0.6*
C16:1n7	1.8 ± 0.3	1.9 ± 0.3	5.0 ± 1.2	6.2 ± 2.2
C18:0	3.7 ± 1.2	1.9 ± 0.8*	4.7 ± 1.1	9.8 ± 3.3*
C18:1n9	14.9 ± 3.9	11.4 ± 2.6	61.1 ± 3.4	43.2 ± 8.4*
C18:2n6	34.0 ± 2.1	42.4 ± 2.3*	12.7 ± 2.0	13.7 ± 1.3
C18:3n3	1.9 ± 0.6	2.2 ± 0.2	1.6 ± 0.2	3.8 ± 1.2*
C20:3n6	0.8 ± 0.2	1.1 ± 0.2	tr.	tr.
C20:4n6	30.7 ± 4.3	27.1 ± 1.6	1.9 ± 0.6	1.9 ± 0.8
C20:5n3	1.5 ± 0.4	1.7 ± 0.5	tr.	tr.
C22:6n3	3.5 ± 0.5	3.7 ± 0.2	tr.	tr.

Data as means ± SD (n = 6). See legend to Table 1S for other details. The asterisk (*) indicates a significant effect of triple-synuclein deficiency when compared with WT ($p < 0.05$).

Table S4 Fatty acid composition of total lipids (% of total fatty acids) in midbrain and cortex from triple-synuclein null mutant (TKO) and wild-type (WT) mice

	MIDBRAIN		CORTEX	
Fatty acid	WT	TKO	WT	TKO
C16:0	16.5 ± 0.2	17.8 ± 1.2	20.9 ± 0.2	21.3 ± 0.6
C16:1n7	0.5 ± 0.1	0.5 ± 0.1	0.5 ± 0.0	0.6 ± 0.1
C18:0	20.6 ± 0.6	20.9 ± 0.4	21.6 ± 0.2	21.2 ± 0.2
C18:1n9	20.1 ± 0.6	19.6 ± 0.9	15.5 ± 0.4	15.5 ± 0.6
C18:1n7	4.9 ± 0.4	4.7 ± 0.3	3.7 ± 0.2	3.7 ± 0.2
C20:1n9	3.2 ± 0.2	2.5 ± 0.3	1.2 ± 0.1	1.2 ± 0.2
C20:4n6	8.3 ± 0.4	9.3 ± 0.4*	10.6 ± 0.2	10.7 ± 0.3
C22:4n6	3.4 ± 0.3	3.3 ± 0.2	2.6 ± 0.3	2.5 ± 0.2
C22:6n3	12.9 ± 0.5	13.6 ± 0.5	17.9 ± 0.3	17.6 ± 0.5
C24:0	0.9 ± 0.1	0.6 ± 0.1*	tr.	tr.
C24:1n9	2.7 ± 0.3	1.8 ± 0.2*	1.1 ± 0.1	1.0 ± 0.3
C26:1n9	2.8 ± 0.2	1.8 ± 0.1*	1.3 ± 0.2	1.3 ± 0.5

Data as means ± SD (n = 6). See legend to Table 1S for other details. The asterisk (*) indicates a significant effect of triple-synuclein deficiency when compared with WT ($p < 0.05$)
